# Multimorbidity among incident Finnish systemic lupus erythematosus patients during 2000–2017

**DOI:** 10.1177/0961203320967102

**Published:** 2020-10-22

**Authors:** Simo Kariniemi, Vappu Rantalaiho, Lauri J Virta, Kari Puolakka, Tuulikki Sokka-Isler, Pia Elfving

**Affiliations:** 1 School of Medicine, University of Eastern Finland, Kuopio, Finland; 2 Department of Medicine, Kuopio University Hospital, Kuopio, Finland; 3 Centre for Rheumatic Diseases, Tampere University Hospital, Tampere, Finland; 4 Faculty of Medicine and Health Technology, Tampere University, Tampere, Finland; 5 Research Department, Social Insurance Institution, Turku, Finland; 6 Terveystalo Healthcare, Lappeenranta, Finland; 7 Faculty of Health Sciences, University of Eastern Finland, Kuopio, Finland; 8 Jyväskylä Central Hospital, Jyväskylä; Finland

**Keywords:** Systemic lupus erythematosus, morbidity, cardiovascular disease, gender, comorbidity

## Abstract

The objective of the study was to examine the risk of other morbidities among patients with systemic lupus erythematosus (SLE). A total of 1006 adult new-onset SLE patients were identified during 1.1.2000- 31.12.2014 from the register of Social Insurance Institution. For each case three general population controls matched according to age, sex and place of residence at the index day were sampled from the population register. Both groups were followed up from the index date until the end of 2017 or until death. The national register on specialized care was explored to gather broadly their 12 organ-specific morbidities, which were found among 91.2% of SLE patients and 66.7% of comparators. The rate ratio (RR) was elevated in almost all disease groups. Musculoskeletal, cardiovascular and genitourinary conditions were the most common comorbidities with RRs of 1.82 (1.68 to 1.97), 1.91 (1.76 to 2.08) and 1.91 (1.73 to 2.09), respectively. Men with SLE had a significantly higher risk for diseases of the genitourinary system and endocrine, nutritional and metabolic diseases compared to women with SLE. The risk of concurrent morbidities is essential to note in the care of SLE patients.

## Introduction

Systemic lupus erythematosus (SLE) is a complex autoimmune disease that can affect almost all organs and tissues. The clinical picture can vary greatly, and it is influenced by gender, age, ethnicity, residence and medication. Due to the heterogeneity of the disease, symptoms can be diverse. Most patients are female.^1–3^

Patients diagnosed with SLE have a considerable burden due to multi-organic involvement of the disease and the treatment chosen for it.^4^,^5^ Previous studies have shown a significant risk for various diseases, such as cardiovascular diseases (CVDs), renal diseases, psychiatric disorders, infections and osteoporosis. For example, the risk for myocardial infarct has been estimated to be 2 to 9 times higher in SLE patients than in the general population.^6–11^

The European League Against Rheumatism (EULAR) recommendations from the year 2008 advise to carefully monitor certain comorbidities, and in recent years, the presence of concurrent disorders in SLE has slowly gained more attention among health care professionals.^12^ Despite the recent publicity, reports on the occurrence of concomitant diseases among patients with SLE are far from ample, and only a few of them depict the wide spectrum of the comorbidities. In this study, we examine broadly the relations between SLE and concurrent diseases.

## Patients and methods

In Finland every resident has national health insurance, and the Social Insurance Institution (SII) keeps a register of them. Patients with chronic inflammatory rheumatic disorders are entitled to a special (higher than basic) reimbursement for the cost of anti-rheumatic drugs. Identification of SLE patients was based on new special reimbursement decisions with the 10th International Classification of Diseases code (ICD-10) of M32 in the register of SII during 1.1.2000 – 31.12.2014. The date of acceptance of reimbursement was defined as the date of diagnosis (index date). For every SLE patient, three individually matched (age, gender and residence at the index date) population controls were selected from the Population Register Centre. Only adults (age >17 years) were included. Rate ratios (RR) were standardized by education level at baseline (basic, middle, lower high and upper high level), and information about education level was acquired from Statistics Finland.

The Finnish law on personal register obligates the service providers to produce information to the Care Register of the National Institute for Health and Welfare (NIHW). The Care Register covers all hospitalizations since 1969. Outpatient visits in specialized care are included since 1998. The data contains, among others, each patient's personal identification code (PIC) and diagnoses of medical disorders according to the codes of the ICD-10.

We retrieved data on 12 organ-specific disease groups and examined some subgroups of special interest as well (Table 1). Systemic connective tissue disorders M30-M36 were excluded from the study and only disease groups of M00-M25 and M40-M99 were included to study from the group of the diseases of the musculoskeletal system and connective tissue. Infectious diseases were not included in this study because the diagnoses made in primary health care would have been missed.

**Table 1.** The number of comorbidities in systemic lupus erythematosus patients and population controls and the rate ratio during 2000–2017 according to 10th revision of the International Classification of Diseases codes.

	ICD-10 disease codes	SLE patientsN = 1006	ControlsN = 3005	RR^a^ (95% CI)	P-value^b^
		N (%)	N (%)		
Malignant neoplasms	C00-D09	117 (11.6)	268 (8.9)	1.29 (1.05 to 1.59)	0.057
Bening neoplasms	D10-D49	201 (20.0)	356 (11.8)	1.68 (1.44 to 1.97)	<0.001
Diseases of the blood and blood-forming organs and certain disorders involving the immune mechanism	D50-D89	178 (17.7)	103 (3.4)	5.15 (4.08 to 6.49)	<0.001
Endocrine, nutritional and metabolic diseases	E00-E90	254 (25.2)	388 (12.9)	1.90 (1.65 to 2.18)	<0.001
Disorders of thyroid gland	E00-E07	101 (10.0)	120 (4.0)	2.49 (1.93 to 3.22)	<0.001
Other hypothyroidism	E03	80 (8.0)	70 (2.3)	3.39 (2.48 to 4.63)	<0.001
Diabetes mellitus	E10-E14	83 (8.3)	138 (4.6)	1.74 (1.34 to 2.27)	<0.001
Disorders of lipoprotein metabolism and other lipidemias	E78	53 (5.3)	98 (3.3)	1.56 (1.13 to 2.16)	0.036
Mental and behavioral diseases	F00-F99	199 (19.8)	399 (13.3)	1.46 (1.25 to 1.70)	<0.001
Dementia in Alzheimer`s disease, vascular dementia, dementia in other diseases classified elsewhere and unspecified dementia	F00-F03	24 (2.4)	72 (2.4)	0.96 (0.61 to 1.52)	0.88
Schizophrenia, schizotypal and delusional disorders	F20-F29	15 (1.5)	40 (1.3)	1.07 (0.60 to 1.93)	0.82
Mood (affective) disorders	F30-F39	102 (10.1)	177 (5.9)	1.71 (1.36 to 2.16)	<0.001
Diseases of the nervous system	G00-G99	313 (31.1)	511 (17.0)	1.78 (1.58 to 2.01)	<0.001
Other degenerative diseases of the nervous system	G30-G32	16 (1.6)	79 (2.6)	0.58 (0.34 to 0.99)	0.14
Epilepsy and status epilepticus	G40-G41	33 (3.3)	34 (1.1)	2.88 (1.79 to 4.63)	<0.001
Diseases of the eye and adnexa	H00-H59	322 (32.0)	499 (16.6)	1.88 (1.67 to 2.12)	<0.001
Iridocyclitis	H20	18 (1.8)	21 (0.7)	2.62 (1.40 to 4.90)	0.015
Diseases of the circulatory system	I00-I99	511 (50.8)	761 (25.3)	1.91 (1.76 to 2.08)	<0.001
Hypertensive diseases	I10-I15	237 (23.6)	351 (11.7)	1.93 (1.67 to 2.24)	<0.001
Ischemic heart diseases	I20-I25	100 (9.9)	177 (5.9)	1.62 (1.29 to 2.04)	<0.001
Cerebrovascular diseases	I60-I69	78 (7.8)	117 (3.9)	1.92 (1.46 to 2.53)	<0.001
Other chronic obstructive pulmonary disease, asthma and status asthmaticus	J44-J46	112 (11.1)	142 (4.7)	2.32 (1.83 to 2.94)	<0.001
Noninfective enteritis and colitis	K50-K52	28 (2.8)	42 (1.4)	2.02 (1.26 to 3.24)	0.021
Disease of the musculoskeletal system and connective tissue	M00-M99^c^	532 (52.9)	863 (28.7)	1.82 (1.68 to 1.97)	<0.001
Osteoporosis with pathological fracture and osteoporosis without pathological fracture	M80-M81	61 (6.1)	35 (1.2)	5.08 (3.38 to 7.64)	<0.001
Diseases of the genitourinary system	N00-N99	456 (45.3)	708 (23.6)	1.91 (1.73 to 2.09)	<0.001
Renal tubulointerstitial diseases	N00-N16	197 (19.6)	94 (3.1)	6.15 (4.86 to 7.78)	<0.001
Renal failure	N17-N19	53 (5.3)	34 (1.1)	4.53 (2.96 to 6.92)	<0.001

^a^Adjusted for education level.

^b^The significance were correct for multiplicity using Hommel’s multiple comparison procedure.

^c^Excluding systemic connective tissue disorders M30–M36; ICD-10 code = 10^th^ International Classification of Diseases code; SLE=Systemic lupus erythematosus; RR = Rate ratio.

The follow-up started from the index date of the each SLE patient and ended when the patient died or at end of the year 2017, whichever occurred first. Permission to use the databases were obtained from the SII and the NIHW. By Finnish law, no approval of an ethical committee nor the patient's informed consent are required for register-based studies done without contacting study subjects.

## Statistical methods

Data are presented as means with standard deviation (SD) and as counts with percentages. Adjusted RRs of comorbidities were calculated using generalized linear models with log link and binomial distribution. Penalized maximum likelihood logistic regression (Firthlogit) was used if the event of interest was rare. Models included education level as a covariate. A possible nonlinear relationship between age at the index day and RR for cardiovascular diseases was assessed by using four-knot-restricted generalized linear models. The length of the distribution (age at the index day) of knots was located at the 5th, 35th, 65th, and 95th percentiles. Knot locations were based on Harrell's recommended percentiles.^13^ Hommel’s adjustment was used to correct levels of significance for multiple testing, because it is more powerful than alternative procedures, including the Bonferroni, Holm's, and Hochberg's procedures.^13^ Stata 16.1 (StataCorp LP; College Station, Texas, USA) statistical package was used for the analyses.

## Results

A total of 1006 patients with newly-diagnosed SLE (mean age 45.5 years, SD 16 years, females 84,0%) and 3005 controls were included. The females were younger than the males: 44.9 years (SD 15.9 years) and 48.6 years (SD 16.4 years), respectively. The cumulative follow-up time was 8631 person years in SLE patients and 26382 person years in controls, with a mean follow-up of 8.6 years and 8.8 years, respectively.

**Figure 1.**
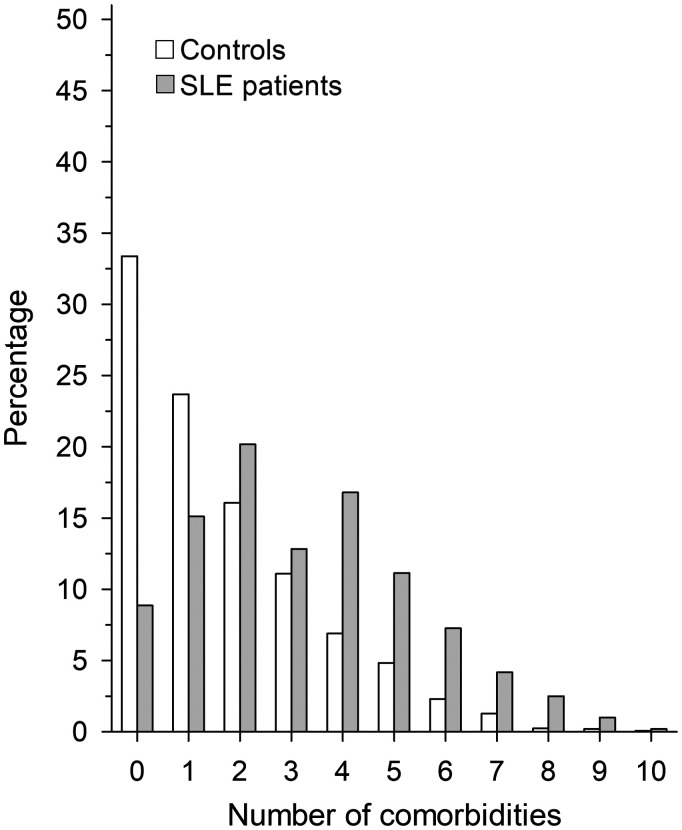
Cumulative number of comorbidities among new-onset systemic lupus erythematosus patients diagnosed between 2000–2014 and their controls at the end of the follow-up 2000–2017. Infections and systemic connective tissue diseases are not included.

**Figure 2.**
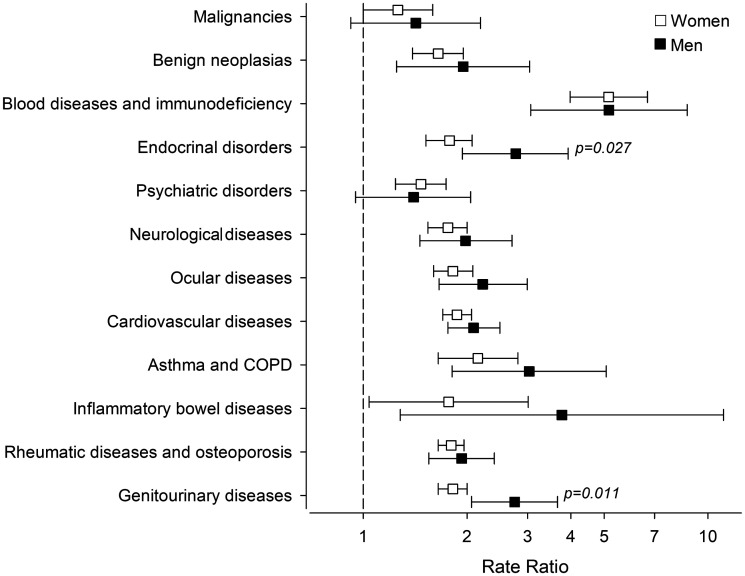
Education level adjusted rate ratios of comorbid diseases of systemic lupus erythematosus patients and controls by gender during 2000–2017 and according to 10th revision of the International Classification of Diseases codes. Malignancies = malignant neoplasms C00-D09, Benign neoplasias = benign neoplasms D10–D49, Blood diseases and immune deficiency = disease of the blood and blood-forming organs and certain disorders involving the immune mechanism D50–D89, Endocrinal diseases = endocrine, nutritional and metabolic diseases E00-E90, Psychiatric disorders = mental and behavioural diseases F00–F99, Neurological diseases = diseases of the nervous system G00-G99, Ocular diseases = diseases of the eye and adnexa H00–H59, Cardiovascular diseases = diseases of the circulatory system I00-I99, Asthma and COPD = other chronic obstructive pulmonary disease, asthma and status asthmaticus J44–J46, Inflammatory bowel diseases = noninfective enteritis and colitis K50–K52, Rheumatic diseases and osteoporosis = disease of the musculoskeletal system and connective tissue M00–M99 (excluding systemic connective tissue disorders M30–M36), Genitourinary diseases = diseases of the genitourinary system N00–N99.

Morbidities of interest were found among 91.2% of SLE patients and among 66.7% of comparators. Musculoskeletal, cardiovascular and genitourinary conditions were the three most common comorbidities in both groups. Number of comorbid conditions per individual was higher among SLE patients (Figure 1). Table 1 displays the numbers of the selected comorbid diseases and the respective RRs. Compared to the general population, SLE patients had elevated RRs for most of the diseases studied. Only schizophrenia, dementia, degenerative diseases of the nervous system and malignant neoplasms were not more frequent in the patient population. Men with SLE had a higher risk for diseases of the genitourinary system and endocrine, nutritional and metabolic diseases (Figure 2). After controlling confounders, no difference was found between genders in the number of comorbidities: men with SLE 3.5 (95% CI: 3.1 to 3.8) and women with SLE 3.2 (95% CI: 3.0 to 3.3); p = 0.10.

**Figure 3.**
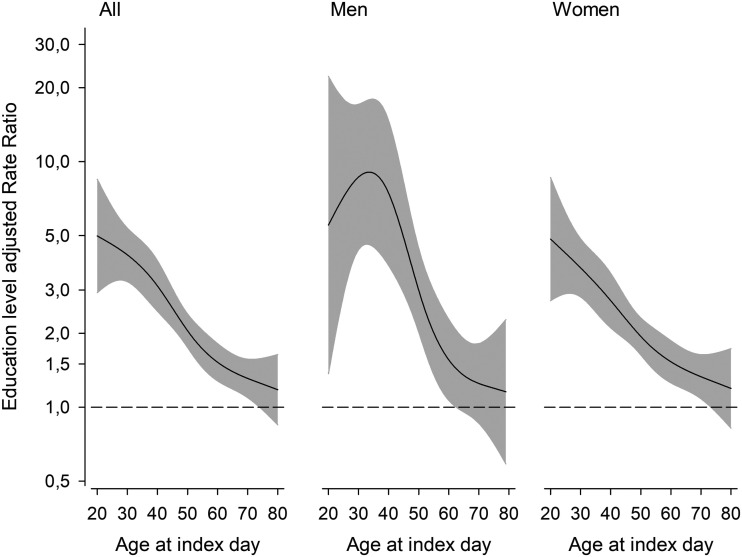
Education level adjusted rate ratios of cardiovascular diseases between systemic lupus erythematosus patients and controls during 2000–2014 according to age at index day. The curves were derived from a four-knot-restricted cubic splines generalized linear models. The models were adjusted for education levels. The grey area represents a 95% confidence interval.

The relative risk of CVDs depended on age. The RR was highest among patients, who were diagnosed with SLE in the young age groups (Figure 3).

## Discussion

In our study, the rate of morbidities was higher in Finnish patients with newly diagnosed SLE than their population controls. Moreover, the number of morbidities per individual was higher.

Our findings are mostly in line with previous research, although some variation in morbidities has been reported depending on study design and ethnicity.^14–16^ In a case-control study from UK SLE patients were compared using general practice records. The risk of comorbidities was elevated in almost all disease groups studied, but especially renal diseases and CVDs were more frequent, as it was in our cohort.^14^ In a South African case-control study including mostly young black women, approximately 80% of SLE patients had more than one comorbidity after a six-year follow-up. However, CVDs, except hypertension, were rare compared to our study or studies performed in other industrial countries.^11^,^15^,^17^

SLE itself predisposes to CVDs due to endothelial dysfunction.^18^,^19^ Again, metabolic syndrome is more frequent in SLE patients, contributing to the CVD burden.^20^ In our study the relative CVD burden was substantial in young men, but young women had increased RR for CVDs as well. With advancing age the CVD risk approached but did not reach that of the general population. This age-related relative risk has also been reported from the UK.^6^

Kidney disease in SLE patients is mostly due to lupus nephritis (LN). The prevalence of LN varies depending on ethnicity, and LN is more common among non-white people.^21^,^22^ Male SLE patients seem to have a greater risk for kidney diseases, and end stage renal disease and renal failure are common among SLE patients.^23^,^24^ Our study results were in line with the aforementioned, although RRs were not so high. Hydroxychloroquinine has been shown to decrease prevalence of chronic kidney disease, and at least in Finland it is often used as the primary medicine in SLE treatment.^25^,^26^ Another explanation for our result may be that almost all of our patients were native Finnish.

SLE affects nervous system inducing neuropsychiatric disorders (neuropsychiatric SLE, NPSLE).^5,27–33^ Mood disorders, and especially depression, are considered to be one of the most prevalent neuropsychiatric comorbidities in SLE.^5^,^34^ Our study results support the aforementioned even though a considerable number of the mild cases are treated in the primary health care and never reach specialized care. However, we found no significant RR for Alzheimer’s disease, vascular dementia, dementia in other diseases classified elsewhere and unspecified dementia or other degenerative diseases of the nervous system.

We found a definite risk for osteoporosis. SLE patients have been shown to be prone to osteoporosis due to systemic inflammation and glucocorticoid treatment.^4^ In addition, vulnerability to osteoporosis may result from sensitiveness to sunlight, lupus nephritis and low D-vitamin levels.^35^,^36^ SLE patients may also be screened for osteoporosis and followed more carefully than other populations.^37^

According to our study, there is a positive correlation between SLE and obstructive pulmonary disease. However, we could not differentiate between asthma and chronic obstructive lung disease (COPD). Conflicting results have been published about the risk of these conditions, and the matter needs more investigation.^38–42^

Men with SLE tend to have more severe disease course than women, at least among white and African-American patients followed in the Hopkins Lupus Cohort. Men more often had cardiovascular, renal and hematological manifestations, whereas women experienced more malar rash, photosensitivity, alopecia, oral ulcers and arthralgia at the end of the follow-up.^43^ In our study, no difference was found between genders in the number of comorbidities, but men with SLE had a higher risk in genitourinary and in endocrine and metabolic diseases.

It is not clear, why SLE seems to be harsher in males. One possible explanation is that men might seek medical care later, which could delay the diagnosis and the start of proper treatment.

A weakness of this register-based study is the lack of clinical data. Therefore, we could not determine the severity of SLE and evaluate whether a harsher disease course was related to a higher morbidity rate. A major limitation in this study is the lack of infectious diseases. In addition, some diagnoses made by general practitioners may be missing. Many SLE patients are regularly monitored in rheumatology outpatient clinics and are more prone to be diagnosed with morbidities than individuals in the general population.

The strengths of this study are the relatively long follow-up time, the case-control study design and the linkage of extensive nationwide information from different official registers. In addition, the diagnoses of morbidities were made in specialized care, strengthening the reliability of the diagnosis. Our study consisted of only new-onset SLE patients and their morbidities manifesting in the following years. This nationwide study included practically all patients using medication for SLE, but some mild forms of the disease without need for disease-modifying anti-rheumatic drugs might have been left out.

In conclusion, our study shows that SLE patients have a considerable burden of various morbidities. Particularly, CVDs are more frequent in SLE patients than in the rest of the population, but vulnerability to other morbidities is also notable.
